# The Other Side of the Coin: May Androgens Have a Role in Breast Cancer Risk?

**DOI:** 10.3390/ijms23010424

**Published:** 2021-12-31

**Authors:** Chiara Chiodo, Catia Morelli, Fabiola Cavaliere, Diego Sisci, Marilena Lanzino

**Affiliations:** Department of Pharmacy, Health and Nutritional Science, University of Calabria, Via P. Bucci, Arcavacata di Rende, 87036 Cosenza, Italy; chiara.chiodo@unical.it (C.C.); catia.morelli@unical.it (C.M.); fabiola.cavaliere@yahoo.it (F.C.); diego.sisci@unical.it (D.S.)

**Keywords:** androgens, dihydrotestosterone, androgen receptor, breast, breast cancer, breast cancer risk

## Abstract

Breast cancer prevention is a major challenge worldwide. During the last few years, efforts have been made to identify molecular breast tissue factors that could be linked to an increased risk of developing the disease in healthy women. In this concern, steroid hormones and their receptors are key players since they are deeply involved in the growth, development and lifetime changes of the mammary gland and play a crucial role in breast cancer development and progression. In particular, androgens, by binding their own receptor, seem to exert a dichotomous effect, as they reduce cell proliferation in estrogen receptor α positive (ERα+) breast cancers while promoting tumour growth in the ERα negative ones. Despite this intricate role in cancer, very little is known about the impact of androgen receptor (AR)-mediated signalling on normal breast tissue and its correlation to breast cancer risk factors. Through an accurate collection of experimental and epidemiological studies, this review aims to elucidate whether androgens might influence the susceptibility for breast cancer. Moreover, the possibility to exploit the AR as a useful marker to predict the disease will be also evaluated.

## 1. Introduction

Breast cancer is the most prevalent female malignancy and the leading cause of cancer death in women, surpassing lung cancer as the most commonly diagnosed cancer, with an estimated 2.3 million new cases (11.7%) worldwide [1,2]. For this reason, breast cancer prevention remains challenging, as an early diagnosis of the disease can lead to a good prognosis and a high survival rate. Although breast cancer screening programs are fundamental in prevention efforts, the introduction of a reliable strategy to predict women at elevated risk and prevent the disease has been less successful [3,4].

Among the molecular markers potentially linked to the risk of developing breast cancer in healthy women [5,6], steroid hormones and their receptors represent good candidates to investigate since they drive the cyclical remodelling of the mammary gland from menarche to menopause [7,8].

Most studies have highlighted the contribution of estrogens and progesterone in breast cancer risk and tumour development. The role of androgens in breast tumour growth, prognosis and treatment has only recently emerged, even though androgen treatment was historically used as an effective therapeutic option for breast cancer. Indeed, for the first half of the 20th century, therapies utilizing androgen receptor (AR) agonists, such as testosterone propionate and fluoxymesterone, were reserved for postmenopausal breast cancer patients, showing a disease regression rate of ~20–25%, which was only slightly less effective than the rate observed following estrogen-based therapy [9,10,11,12,13]. However, the occurrence of adverse side-effects, such as increased aggressive behaviour and virilisation [10], together with the ability of androgens to be converted into estrogens, caused the loss of androgen therapy popularity in the 1970s. Concomitantly, Selective Estrogen Receptor Modulators (SERMs) became the primary treatment option due to a better efficacy and toxicity profile over conventional hormonal therapies [11,12].

AR expression and action differ among the various breast cancer subtypes. The AR shows a higher positivity in estrogen receptor alpha-positive (ERα+) luminal A tumours than in triple negative breast cancer (TNBC) [14,15,16]. Moreover, the AR acts as a tumour suppressor in the diverse setting of ERα+ breast cancer, including endocrine-resistant cancers. Indeed, the AR is associated with favourable clinicopathological features and a better outcome in this subset of cancers and represents a positive predictive biomarker of the response to endocrine therapy [14,17,18]. AR agonists, such as the natural ligand dihydrotestosterone (DHT) or the Selective Androgen Receptor Modulator (SARM) enobosarm (lacking virilising activity), exert an anti-proliferative effect in normal mammary epithelium, as well as in ERα+ breast cancer, both in vitro and in vivo [18,19,20,21,22,23,24,25,26]. This observation strengthens the idea of using SARM for novel androgen-targeted therapy in ERα-driven breast cancers.

On the other hand, the role of the AR in ERα negative (ERα-) breast cancer is controversial and its prognostic value remains uncertain [27,28,29]. Androgen administration has been shown to exert an anti-proliferative effect in ERα- breast cancer cell lines through the AR-mediated up-regulation of the tumour suppressors PTEN, which, in turn, by inducing p53 and p73 expression, promotes apoptosis [30,31]. Nevertheless, AR signalling is mainly considered oncogenic in these tumour settings [8,32,33]. In fact, AR activation controls cell cycle progression by increasing cyclin D1 expression while reducing p73 and p21 levels [34], and promotes epithelial–mesenchymal transition, migration and invasiveness in AR+ TNBC cell lines. Accordingly, blocking AR activity and synthesis reduces tumour growth in patient-derived xenograft models [35,36]. Since a similar pro-tumorigenic role of AR has also been observed in ERα-/HER2-positive (HER2+) breast cancers [37], AR blockage has been proposed as an effective treatment strategy in ERα- breast cancer [38,39,40].

Therefore, the heterogeneity of androgen action within the different breast cancer subtypes has driven the chance to exploit specific AR signalling pathway(s) for successful therapeutic interventions in ongoing clinical trials, employing both AR agonists and antagonists for the treatment of different cohorts of breast cancer patients (Table 1) [41].

Despite this well-documented importance of AR signalling in breast cancer growth and progression, very little is known about the association of androgens, AR expression and breast cancer risk in healthy individuals. This review aims to elucidate whether AR activity might impact on breast cancer susceptibility by collecting evidences of AR-dependent signalling in normal breast epithelium and on its relationship with well-known breast cancer risk factors.

## 2. AR Expression and Action in Normal Mammary Gland

Normal mammary epithelium arises from three specific subsets of cells, hierarchically classified in one population of basal and two populations of luminal mammary epithelial cells. The latter are identified as secretory or hormone-sensing cells and also named as L1 and L2, respectively. L1 secretory luminal mammary epithelial cells are mainly involved in milk production and are characterized by the lack of both ER and progesterone receptors (PR); L2 hormone-sensing mammary epithelial cells, instead, express ER and PR and have a crucial role in the endocrine stimulation of the gland [42,43]. Indeed, receptor-positive cells are the sensors of local and/or circulating steroid hormones, which regulate mammary epithelium differentiation through autocrine and/or paracrine signals [44].

Interestingly, within the normal human breast tissue, AR has been found to be expressed in both basal and, to a greater extent, in hormone-sensing mammary epithelial cells [42,43]. This concomitant expression of the AR in both basal and hormone-sensing cell subpopulations may explain the dichotomous role exerted by the AR in the different breast cancer settings [42,45]. It has been demonstrated that the AR is the most expressed nuclear receptor in both human and mice ERα+/PR+ mammary luminal epithelium [8,46]. More specifically, the AR has been suggested to sustain a luminal phenotype, as evidenced by its expression within cells undergoing a basal-to-luminal transition. To explain how this occurs, a model has been proposed in mice, in which expression and activation of the AR in basal mammary epithelial cells fosters the achievement of a luminal trait [42] in terms of morphology, position within the mammary gland and gene expression [42,43,47]. This model is supported by the observation that AR signalling is also involved in supporting the maintenance of a luminal state in the human breast cancer framework [48]. Thus, the AR-dependent signalling operates in both basal and luminal mammary epithelial cells. The AR promotion of a basal-to-luminal differentiation in mammary epithelial cells may have a fundamental role in normal breast physiology. Indeed, since testosterone, the main female circulating androgen [8], is the preeminent estradiol precursor [49], it has been speculated that ligand–AR activation may favour a luminal phenotype by acting as an indicator of a sufficient estrogen availability to maintain the ER/PR crosstalk necessary for mammary gland differentiation and function [42].

Thus, the AR-dependent signalling operates in both basal and luminal mammary epithelial cells. Findings regarding its participation in the mammary gland developmental process are controversial.

Gao et al., observed a significant increase in ductal growth and extension and a higher number of terminal end buds, (corresponding to human terminal duct luminal units, TDLUs) [50] in AR-knockout (AR^ex3Δ^KO) female mice at the pubertal time, compared to wild-type females. The latter, instead, responded to DHT administration with a ~50% reduction in ductal extension [51]. Oddly, in adult AR^−/−^ mice after puberty, a reduced ductal branching was observed. This condition was partially restored during pregnancy, but the number of milk-producing alveoli remained smaller [52].

In the human breast, androgens mainly exert anti-proliferative effects under physiological conditions. In males, when the estradiol (E_2_)–testosterone (T) ratio is altered due to estrogen excess and/or androgen deficiency, gynecomastia can occur [53,54,55]. Indeed, about 70% of men affected by Klinefelter syndrome, a sex chromosome disorder associated with hypogonadism, display gynecomastia and have an increased risk of developing a breast tumour [56].

In females, breast changes during the lifetime phases, puberty, menstrual period and menopause, arise from the dynamic interplay of androgens and estrogens [57]. In preclinical studies conducted on ovariectomized rhesus monkeys, T co-administration with E_2_, notoriously mitogenic for mammary cells, caused a 40% inhibition of the E_2_-induced expression of the epithelial cell proliferation marker Ki67. This effect occurred through the reduction of ERα expression/activity [58], suggesting that androgens act as modulators of estrogen effects on mammary epithelial cell proliferation. Analogously, in the human female at puberty, androgens reduce breast growth by inhibiting ductal elongation and the proliferation of epithelial cells, antagonizing the stimulatory effects of estrogens [46]. Of note, the androgen anti-proliferative and anti-estrogenic activity is not limited at puberty but is maintained during adulthood [57], suggesting the crucial role of AR/androgen signalling in modulating lifetime breast development and changes.

## 3. Androgen Signalling in Normal Breast Epithelium and Cancer Susceptibility

The hierarchical organization of normal mammary epithelium, and the molecular markers expressed within, are promising keys to understand the origin of breast cancer and to prevent its occurrence [43,45]. Since the majority of mammary tumours show hallmarks of a luminal phenotype, including steroid receptor positivity [59], it should not be a surprise that their distribution in normal breast tissue might strongly dictate cancer susceptibility. Although the AR is recognized as an important player in mammary gland physiology, and it is considered to be an emerging prognostic marker in ER-positive breast cancers [60,61], data regarding the relationship between AR expression and activity in mammary epithelial cells and cancer risk are still very limited.

The potential correlation between AR structure and breast cancer susceptibility has been investigated in a number of studies. Exon 1 of the *AR* gene carries a short tandem of 8 to 35 glutamine CAG repeats, whose length seems to inversely correlate with AR transcriptional activity. In fact, the shorter the AR-CAG unit length is (<19 repeats), the higher the AR activation [62,63,64]. Thus, the CAG polymorphism can lead to receptor dysfunction and, consequently, to pathological conditions such as androgen insensitivity [65,66] and can affect the growth of androgen-dependent tumours, such as prostate cancer [67].

These evidences suggested investigating if the CAG length could also impact on breast cancer risk. A potential increased risk was evidenced by Haiman et al., among Caucasian women with long CAG repeats only with a concomitant first-degree breast cancer family history [68]. However, additional study population and epidemiological analysis revealed a positive [69,70,71] as well as a negative association [64] between the longest AR-CAG units and an increased risk of developing the disease. Unfortunately, the conflicting data available, mainly due to age, ethnicity or family history patient variability, do not provide the possibility to establish an effective association between abnormal AR structure, and subsequent AR activity, and breast tumour susceptibility.

As stated above, characterization of the hierarchical organization and function of mammary epithelial cells is crucial for a better comprehension of how breast cancer arises as well as for the identification of novel breast cancer risk biomarkers [43,45]. In this context, examination of a very large number of human breast tumours indicated that expression of the AR in normal luminal mammary epithelial cells did not overlap with the expression of Ki67 [72], a well-known marker of cell proliferation strongly correlated with breast cancer risk [6,73], indicating a lower proliferating profile for this cell subtype [72].

More recently, the possibility of using the AR as a predictor marker of breast cancer risk was investigated in a nested case-control study on women with benign breast disease (BBD). In the analysed population (78 patients vs 276 controls), no associations were found between AR expression in normal human mammary tissue, evaluated by immunohistochemistry on BBD biopsies, and breast cancer risk. However, when AR expression was assessed in relation to ERα (47 patients vs 127 controls), high levels of AR were moderately associated with a lower risk of disease [74]. Unfortunately, although confirming the ability of AR to antagonize ERα activity [75,76,77], the small sample size used in this study does not allow a relationship between AR and ERα expression in normal mammary epithelium and cancer susceptibility to be established.

**Figure 1:**
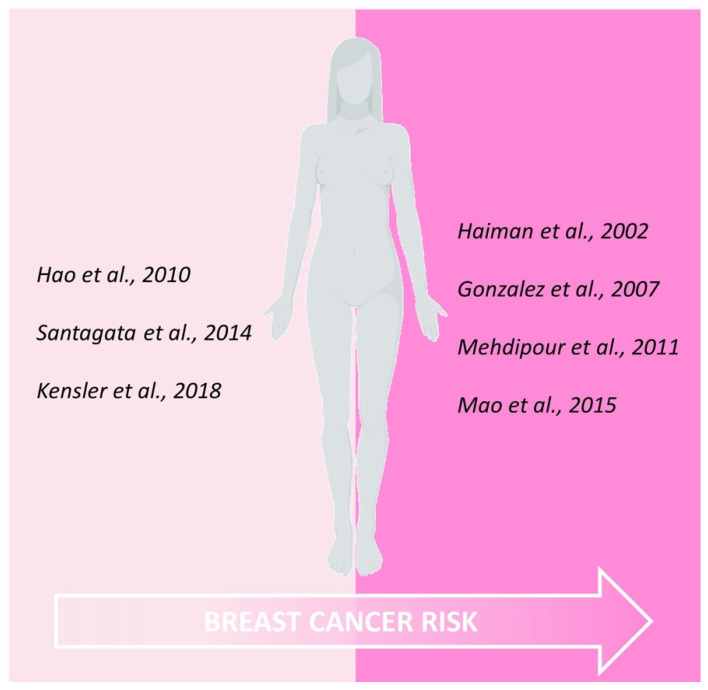
Schematic representation of current evidences correlating AR expression within human normal epithelial cells and subsequent breast cancer risk.

Therefore, even if the available findings (Figure 1) seem to suggest a protective role of AR in preventing breast cancer onset, due to the very limited data at one’s disposal, future investigations are needed to better clarify the specific role of AR in the crucial events triggering a neoplastic transformation in mammary epithelial cells.

## 4. Exogenous Androgen Exposure and Breast Cancer Incidence

Androgens have often been used for therapeutic purposes in women, mainly to treat hypoactive sexual desire disorder or menopausal symptoms [78,79]. Therefore, the evidence of an anti-proliferative action of androgens in normal mammary cells raises the question of what their impact would be on breast cancer risk and development.

The effects of T exposure on breast cancer development have been recently investigated in a prospective 10 year cohort study enrolling 1267 pre- and postmenopausal women receiving implants of T to treat the symptoms of androgen deficiency or T implants in combination with the aromatase inhibitor anastrozole (a therapeutic option for estrogen excess, obesity and breast cancer prevention). Invasive breast cancer (IBC) was diagnosed in 11 women, while 3 patients developed ductal carcinoma in situ (DCIS). Interestingly, comparing the results with the age-matched Surveillance, Epidemiology, and End Results (SEER) Program, used for the assessment of breast cancer incidence rates, showed that the incidence of both IBC and DCIS was lower in the examined population (165/100,000 p-y vs 271/100,000 p-y for IBC; 45/100,000 p-y vs 84/100,000 p-y for DCIS) [80]. Accordingly, a significant reduction in IBC risk, compared to the age-matched SEER incidence rate, was recently evidenced in pre- and postmenopausal women following T or T + E_2_ implant therapy [81]. However, no association was found between the use of transdermal T for the treatment of the hypoactive sexual desire disorder in postmenopausal women and an increased incidence of breast cancer [82].

This protective effect of exogenous androgens is consistent with previous studies, which detected a lower proliferative index in mammary tissues from women undergoing estrogen–progestin hormone replacement therapy (HRT) in combination with T, compared to HRT alone [83], as well as in ex vivo normal human breast tissues exposed to DHT [84,85].

Nevertheless, these observations are in contrast with the findings that contraceptive progestins with androgenic activity, such as the T-related and widely used levonorgestrel, promote hyperproliferation of the breast epithelium with cytological changes, in a xenograft model obtained through intraductal injection of human mammary cells expressing hormone receptors [86]. Though limited by the fact that the hormonal milieu of human breast epithelial cells in mice is quite different to that in humans, these findings are consistent with a prospective study, involving 1,359,323 p-y from 1978 to 2002, that revealed an increased risk of IBC in postmenopausal women receiving estrogen/androgen hormone therapies compared with patients treated with estrogens alone [87].

A good tool to investigate the potential correlation between exogenous sex steroid hormones and breast cancer susceptibility is provided by transgender patients since androgen therapy is crucial to achieve the gender switch through physical feature changes in trans men with female sex assigned at birth. According to androgens’ anti-proliferative properties on breast epithelial cells, T therapy reduces mammary gland volume, increases fibrous connective tissues and inhibits the incidence of breast cancer in transgender men [88,89]. However, it is worth underlining that, in these patients, the disease is rare and only documented by a few case reports evaluating AR expression in tumour tissue [90,91,92]. Tanini et al., reported one case of IBC and one of DCIS in two trans men patients, both before top surgery. The diagnosis occurred following 3 and 2.5 years of T therapy, and the AR expression was 60% and 80%, respectively. Nevertheless, both patients reported a strong breast cancer family history [93], which is often a limiting factor in the evaluation of androgen therapy impact on breast cancer risk in this subset of patients.

Another recent retrospective cohort study investigated the consequence of hormone therapy in 3489 Netherland transgender (2260 trans women and 1229 trans men). Only four cases of IBC were diagnosed in trans men, who were associated with a high risk, compared to cisgender men, and with a lower risk, compared to cisgender women [88].

Thus, the role of exogenous androgens in preventing or promoting breast cancer is still controversial. However, available data suggest that androgen administration might be likely to be associated with a reduced or, at least, unchanged risk of developing breast cancer.

## 5. Androgen Over-Production and Breast Cancer Risk

Androgen over-production may occur in para-physiological and pathological conditions (i.e., polycystic ovarian disease, adrenocortical carcinoma, ovarian hyperthecosis, adrenal hyperplasia) [94]. In such circumstances, for the androgen excess theory of breast cancer [95], androgen surplus is regarded as the key player of endocrine imbalance in women with breast cancer due to the lack of proper control on the mammary epithelial growth and the creation of a hormonal vicious circle. Indeed, a prolonged exposure to high levels of T leads to a constant increase in estradiol production by the aromatase enzyme, resulting in ERα synthesis induction and a continuous cell proliferation. Concomitantly, the conversion of T into its non-aromatizable metabolite DHT by the 5α-reductase enzyme, is also enhanced. At first, increased DHT levels exert their anti-estrogenic and growth inhibitory effects but, in the long run, they are unable to block estrogen proliferative action, unless the source of androgen excess is removed [57].

Accordingly, increased prepubertal androgen levels have been associated with earlier breast development [96].

Houghton et al., recently reported that androstenedione, T and free-T (fT) levels were higher in girls with a first-degree breast cancer family history (BCFH) than in those without a BCFH, while dehydroepiandrosterone sulfate (DHEA-S) was increased only in girls with breast cancer-specific distress, presumably following adrenal gland hypersecretion due to the hypothalamic–pituitary–adrenal axis activation. Importantly, since DHEA-S is not only a natural precursor of androstenedione and T but also of E_2_, earlier breast development might be likely to be linked to estrogen production. Thus, these patients may also be considered at high risk of developing breast cancer [96].

Increased levels of androgens can also result from ovarian stromal hyperplasia. Therefore, a proposed therapeutic approach in postmenopausal women with excessive ovarian androgen production and at high risk of developing breast cancer consists of reducing high T blood levels by administrating gonadotropin-releasing hormone analogues, leading to a medically-induced oophorectomy: lowering T serum concentrations means losing substrate for estrogen synthesis and, in turn, depriving E_2_ proliferative stimulus on breast cells [97].

The positive association between high endogenous androgens’ serum levels and breast cancer risk has been confirmed by several other studies, both in pre- [98,99,100] and postmenopausal women [101,102].

Conclusively, being a source of estrogens, androgens’ hyperproduction seems to correlate to an increased risk of breast cancer. However, since most of the studies evaluated circulating T levels that are metabolized in estrogens [103] and the effects of T on mammary cell proliferation are closely related to 5α-reductase and aromatase tissue expression and activity, these evidences cannot totally reflect the hormone concentration within normal breast tissue because the gland follows its own specific intracrinology [32]. Therefore, a promising method to measure breast steroid hormone levels could be the fine nipple aspiration that, allowing to distinguish the differences between local and serum hormone concentrations [104,105], might provide helpful data to assess breast cancer susceptibility. Moreover, AR expression was not assessed in these studies, making it difficult to understand if AR activation might promote, or not, the malignant transformation.

## 6. Androgens/AR and Breast Cancer Risk Factors

Several factors contribute to a woman being considered at high risk of developing breast cancer, including family history, high-risk predisposition genes, high mammographic density (MD) and Single Nucleotide Polymorphisms [106]. MD has been indicated as a strong and independent risk factor for breast cancer [107,108]. However, few studies have evaluated the association of endogenous sex steroids with MD and breast cancer risk [106,109]. Regarding androgens, endogenous serum fT has been inversely associated with MD in premenopausal women [110]. In addition, circulating dehydroepiandrosterone counteracts fT effects on MD in healthy postmenopausal women by antagonizing its binding to the AR [111,112].

An important correlation also exists between germ-line mutations in breast cancer susceptibility genes 1 and 2 (BRCA1 and BRCA2), which are associated with a 40–85% lifetime breast cancer risk [106], and AR structure and transcriptional activity. The AR polymorphism characterized by longer AR-CAG repeats within the *AR* gene and resulting in a deficient AR activity, has been associated with an increase in breast cancer risk at an early age in patients carrying the BRCA-1 mutation [113]. Of note, BRCA-1 overexpression is able to overcome the AR-CAG repeats-driven inhibitory effect on AR activity in mammary and prostate epithelial cell lines. Indeed, overexpressed BRCA-1 and the p160 coactivator GRIP1, synergically coactivate AR, increasing its transcriptional activity [114].

## 7. Conclusions

In the struggle to defeat breast cancer, prevention programs play an essential role, even long before disease onset: valuable breast cancer risk markers and prediction models, by improving individualized risk assessment, could be helpful, not only for the patients but also for their health providers, in making decisions about the right screening program and/or chemoprevention procedure to adopt.

Endogenous sex hormones’ levels have been associated with increased breast cancer risk. Due to the pivotal role of estrogens in normal breast proliferation and the identification of increased ERα expression as a risk factor for breast cancer development, preventive therapy is aimed at reducing estrogen levels and/or antagonizing receptor activity.

On the other hand, very few reports focus on the potential role of androgens and AR status as markers of breast cancer risk in healthy individuals, and this review was meant to cope with this aim.

Nevertheless, given the complexity of AR action within the breast, no univocal conclusion could be drawn. Indeed, the available data on the role of androgens/AR in preventing or promoting breast cancer are still controversial. In fact, AR-dependent signalling has been reported to exert either beneficial or deleterious effects on breast tissue, even if, under physiological conditions, its activation seems to protect against breast cancer development, at least in part for its ability to counteract ERα action.

These discrepancies are mostly due to the lack of normal breast sample availability and non-uniform cohorts of patients recruited for clinical studies (e.g., patients previously subjected to other treatments and/or their breast cancer susceptibility), as well as due to the complexity of androgen intracrinology within the mammary gland.

Therefore, future studies are needed to accurately discern the role of androgens/ARs in normal breast tissue, so as to unambiguously understand whether the receptor might become a useful and reliable biomarker in breast cancer preventive screening programs. As happened for ERα, this understanding will shed light on the possible exploitation of SARMS or antagonists as novel potential chemoprevention agents in women with a high risk of breast cancer.

## Figures and Tables

**Table 1:** Current clinical trials (>100 participants) investigating the safety and the efficacy of AR agonists or antagonists in women affected by breast cancer (https://clinicaltrials.gov/, accessed on 22 July 2021).

Identifier	Title	Phase	Treatment	Activity	Disease
NCT02463032	A Phase 2 Open Label, Multi-Center, Multinational, Randomized, Parallel Design Study Investigating The Efficacy and Safety Of GTx-024 On Metastatic or Locally Advanced ER+/AR+ Breast Cancer (BC) in Postmenopausal Women	2	GTx-024	Selective-AR modulator	ER+ AR+ metastatic or locally advanced BC
NCT02007512	A phase 2, randomized, double-blind, placebo-controlled, multicenter study of efficacy and safety of enzalutamide in combination with exemestane in patients with advanced breast cancer that is estrogen or progesterone receptor-positive and her2-normal	2	Enzalutamide/ Exemestane/ Placebo	AR inhibitor/ Aromatase inhibitor	ER+ or PR+ and HER2 normal advanced BC
NCT01889238	A phase 2, single-arm, open-label, multicenter study of the clinical activity and safety of enzalutamide in patients with advanced, androgen receptor-positive, triple-negative breast cancer	2	Enzalutamide	AR inhibitor	AR+ TNBCadvanced BC
NCT03650894	A Phase II Study of Nivolumab Combined With Bicalutamide and Ipilimumab in Metastatic HER2-negative Breast Cancer	2	Nivolumab/ Ipilimumab/ Bicalutamide	anti-PD-1/ anit-CTLA4/ AR inhibitor	HER 2- metastatic or unresectable BC
NCT02091960	A Phase 2, Multicenter, Open-label Study to Assess the Efficacy and Safety of Enzalutamide With Trastuzumab in Subjects With HER2+ AR+ Metastatic or Locally Advanced Breast Cancer	2	Enzalutamide/ Trastuzumab	AR inhibitor/ HER2 inhibitor	AR+ HER2+ metastatic or locally advanced BC
NCT00725374	An Exploratory, Double-blind, Randomized, Placebo-controlled Trial to Investigate the Tissue Specific Effects of 2.5 mg Tibolone on Breast Cancer in Postmenopausal Women, in Particular on Breast Tissue Proliferation	3	Tibolone/ Placebo	Selective-ER modulator	BC
